# Reward Motivation Adaptation Deficits Are Specific to Co-Occurring Subclinical Depression and Anhedonia

**DOI:** 10.3390/bs16030464

**Published:** 2026-03-20

**Authors:** Xin Gao, Jie Pu, Xinyue Zhao, Yuxi Zhao, Wenting Mu, Simon S. Y. Lui, Jia Huang, Raymond C. K. Chan

**Affiliations:** 1 Neuropsychology and Applied Cognitive Neuroscience Laboratory, State Key Laboratory of Cognitive Science and Mental Health, Institute of Psychology, Chinese Academy of Sciences, Beijing 100101, China; gaox@psych.ac.cn (X.G.); puj@psych.ac.cn (J.P.); zhaoxinyue@psych.ac.cn (X.Z.); zhaoyuxi24@mails.ucas.ac.cn (Y.Z.); rckchan@psych.ac.cn (R.C.K.C.); 2 Department of Psychology, University of Chinese Academy of Sciences, Beijing 101408, China; 3 Sino-Danish College, University of Chinese Academy of Sciences, Beijing 100049, China; 4 Department of Psychological and Cognitive Sciences, Tsinghua University, Beijing 100084, China; wmu@mail.tsinghua.edu.cn; 5 Neuropsychology and Applied Cognitive Neuroscience Laboratory, Department of Psychiatry, School of Clinical Medicine, The University of Hong Kong, Hong Kong Special Administrative Region, China; lsy570@hku.hk

**Keywords:** reward motivation adaptation, subclinical depression, effort–reward imbalance, anhedonia

## Abstract

Reward motivation adaptation is defined as the extent to which the willingness to exert effort varies as a function of incentive salience, encompassing both motivational (‘wanting’) and hedonic (‘liking’) components. Although reduced reward motivation has been reported in subclinical depression and anhedonia, it remains unclear whether impaired adaptation is a general feature of subclinical depression or is more evident when depressive symptoms co-occur with anhedonia. We addressed this question in two behavioral studies using a task that systematically varied effort–reward ratios. Study 1 contrasted three screening-based groups: individuals with elevated social anhedonia, individuals with subclinical depression without high social anhedonia, and controls with low levels of both, and found no clear group differences in reward motivation adaptation across effort–reward conditions. Study 2 focused on female participants with subclinical depression who also showed higher levels of anhedonia, compared with non-depressed controls. In this sample, the subclinical depression group showed lower overall reward motivation and indications of reduced ‘liking’ adaptation. In conclusion, these findings suggest that deficits in reward motivation adaptation were not clearly observable when subclinical depression or social anhedonia were considered in isolation, but may emerge when depressive status and broader measures of anhedonia co-occur, though this pattern requires confirmation in larger and more diverse samples.

## 1. Introduction

Reward motivation is defined as an individual’s willingness to exert effort to obtain rewards, as reflected in motivational ‘wanting’ and hedonic ‘liking’ components (Berridge et al., 2009; Berridge & Robinson, 2016; Yan et al., 2024). In daily life, reward motivation is dynamic rather than static, fluctuating in response to changing environments. Reward motivation adaptation refers to the flexible adjustment of effort expenditure as a function of varying effort–reward contexts (Yan et al., 2024). Previous studies have reported reduced reward motivation in individuals with major depressive disorder (MDD) (Treadway & Zald, 2011; Horne et al., 2021) and in those with subclinical depression (Yang et al., 2014). Such reward-related dysfunctions are also core features of anhedonia (Beevers et al., 2013; Eshel & Roiser, 2010; Halahakoon et al., 2020; Pizzagalli, 2022; Treadway et al., 2009; C. Wu et al., 2025). However, few studies have ever explored whether these depressive conditions impede the ability to dynamically regulate effort for reward as contexts shift.

Subclinical depression, or subthreshold depression, generally refers to clinically relevant but attenuated depressive symptoms that do not meet the full diagnostic criteria for major depressive disorder (Judd et al., 1994; R. Zhang et al., 2023). Although symptoms fall below diagnostic thresholds, subclinical depression is associated with impaired functioning and poorer quality of life compared to non-depressed individuals (Rodríguez et al., 2012), and longitudinal evidence further indicates that it is highly prevalent in the general population and confers elevated risk for subsequent major depression (R. Zhang et al., 2023).

Within the subclinical depressive symptoms, reduced pleasure experience and motivation are considered especially relevant for reward processing. Anhedonia, defined as a reduced capacity to experience pleasure (Feighner, 1972), is widely considered a core symptom domain closely linked to reward-related dysfunction (Höflich et al., 2019; Treadway & Zald, 2011; C. Wu et al., 2025). Social anhedonia, which refers to diminished pleasure derived from interpersonal interactions, whereas broader measures of anhedonia extend to non-social domains (Q. Wu et al., 2020). Social anhedonia or other broader measures of anhedonia all relate to the altered processes, such as expectation, valuation and effort allocation, in depressive conditions (Solomonov et al., 2023). Thus, anhedonia features, including social anhedonia, are a plausible contributor to individual differences in reward motivation adaptation within subclinical depression.

Within reward processing theories, ‘wanting’ and ‘liking’ are separable components that may be differentially affected by anhedonia (Berridge et al., 2009; Berridge & Robinson, 2016). In this framework, anhedonia is often discussed in terms of reduced anticipatory and consummatory pleasure, which are commonly linked to ‘wanting’ and ‘liking’, respectively (Treadway & Zald, 2011). Reduced anticipatory pleasure has been associated with lower willingness to expend effort for reward, partly through altered expectations of future reward value and distorted cost–benefit appraisal (Chentsova-Dutton & Hanley, 2010; Frey et al., 2023; Horne et al., 2021; McFarland & Klein, 2009; Sherdell et al., 2012; Yang et al., 2014), whereas reduced consummatory pleasure may be more closely related to diminished experienced reward value once the reward is obtained (Sherdell et al., 2012). These findings support an association between anhedonia and static deficits in reward motivation or pleasure. Much less is known about whether individuals with subclinical depression and anhedonia features can adaptively increase ‘wanting’ and ‘liking’ when the effort–reward ratio becomes more favorable. This question calls for paradigms that directly manipulate effort–reward conditions and assess changes in both components.

To quantify reward motivation adaptation across different effort–reward ratios, Yan et al. (2024) developed the Reward Motivation Adaptation Task (RMAT), which operationalizes reward motivation adaptation by systematically varying the effort–reward ratio and measuring how motivation changes across these contexts. RMAT assesses both ‘wanting’ and ‘liking’, allowing these components to be examined as the effort–reward context shifts (Yan et al., 2024). The task structure conceptually mirrors effort–reward imbalance (ERI), where high effort with low reward undermines motivation and increases strain (Siegrist, 1996; Studer & Knecht, 2016). Initial work using this paradigm suggests that it is sensitive to individual differences in context-dependent reward motivation (Yan et al., 2024), but it remains unclear whether similar adaptation difficulties are observed in individuals with subclinical depression and anhedonia-related dysfunction.

A further issue concerns how anhedonia is conceptualized and operationalized in the present work. Anhedonia is a multidimensional construct that can be assessed as relatively specific trait dimensions, such as social anhedonia, or as broader symptom burden across reward domains (Case et al., 2022). In the present research, we used a two-study design to address these levels from complementary perspectives within subclinical depression. Study 1 focused on social anhedonia indexed by the Chapman Social Anhedonia Scale (CSAS; Chapman et al., 1976) and contrasted individuals with high social anhedonia, individuals with subclinical depression without high social anhedonia, and healthy controls. Study 2 compared a subclinical depression group and non-depressed controls who differed on questionnaire measures related to pleasure experience and motivation. Accordingly, in this article, we use the term anhedonia at the construct level, while Study 1 and Study 2 distinguish between social anhedonia as a trait and broader anhedonia-related impairment in subclinical depression. In addition to this difference in how anhedonia was operationalized, Study 2 also administered the RMAT in a controlled laboratory setting rather than online, in order to reduce environmental distractions and shorten the delay between symptom screening and task completion. Study 2 was restricted to women as a pragmatic and exploratory decision to reduce gender-related heterogeneity in an initial extension of the paradigm and to focus on a subgroup in which depression is especially common, given that women show approximately twice the prevalence of depression compared with men in epidemiological studies (Salk et al., 2017).

Based on this framework, we formulated specific hypotheses for each study. In Study 1, we expected that individuals with high social anhedonia and those with subclinical depression without high social anhedonia would show lower reward motivation than healthy controls. We also anticipated that these two subclinical groups would show less pronounced changes in wanting and liking across different effort–reward contexts, indicating reduced flexibility in reward motivation adaptation. In Study 2, we expected that individuals with subclinical depression would, relative to non-depressed controls, show lower overall levels of ‘wanting’ and ‘liking’ and weaker modulation of these indices across effort–reward conditions as they became more or less favorable.

## 2. Study 1

Study 1 used an online behavioral reward motivation adaptation task that systematically manipulated effort–reward ratios to examine the adaptation of reward motivation. Participants were assigned to one of three groups based on screening measures: a high level of social anhedonia group (HSA), a subclinical depression without high level of social anhedonia group (SD-noHSA), and a healthy control group (HC). The hypothesis was that individuals in the HSA group and SD-noHSA group would show deficits in adapting their reward ‘wanting’ or ‘liking’ with different effort–reward ratios.

### 2.1. Participants

A priori power analysis was conducted using G*Power 3.1.9.7 (Faul et al., 2009) for a mixed-design repeated-measures ANOVA with a within–between interaction. Assuming a medium effect size (Cohen’s *f* = 0.25), *α* = 0.05, and power (1 − *β*) = 0.80, with three groups and three repeated measurements, the estimated minimum total sample size was 36 (with the default settings of correlation among repeated measures = 0.50 and nonsphericity correction *ε* = 1.00).

A total of 192 college students were recruited from several universities in China through posters and advertisements, with data collected from September 2022 to April 2023. The inclusion and exclusion criteria for each of the three groups are as follows: HSA group: (1) CSAS score ≥ 20 (R.-T. Zhang et al., 2025); (2) PHQ-9 scores < 10; (3) aged 18–30 years. SD-noHSA group: (1) PHQ-9 score ≥ 10 (Kroenke et al., 2001); (2) CSAS scores < 20; (3) aged 18–30 years. HC group: (1) did not meet criteria for other groups; (2) aged 18–30 years. Exclusion Criteria: Participants were excluded if they: (1) were taking psychotropic medications; (2) had a diagnosis or history of other mental disorders; (3) had a history of substance dependence or abuse; (4) had a history of brain injury or neurological disorders; (5) had a family history of mental illness.

### 2.2. Measures

#### 2.2.1. The Chinese Version of the Patient Health Questionnaire (PHQ-9)

The PHQ-9 is a self-report scale developed by Kroenke et al. (2001) for assessing depressive symptoms. W. Wang et al. (2014) translated the scale into Chinese and confirmed its reliability. The Chinese version consists of nine items that assess respondents’ depressive symptoms over the past two weeks using a four-point Likert scale (0: not at all, 3: almost every day).

#### 2.2.2. The Chinese Version of the Chapman Social Anhedonia Scale (CSAS)

The CSAS was developed by Chapman et al. (1976) to measure individuals’ ability to experience pleasure in social situations. It was translated into Chinese by Chan et al. (2012b) and was shown to have good reliability. The Chinese version includes 40 true/false items, with higher scores indicating greater difficulty in experiencing pleasure in social situations.

#### 2.2.3. The Chinese Version of the Snaith–Hamilton Pleasure Scale (SHAPS)

The SHAPS was developed by Snaith et al. (1995) to assess an individual’s ability to experience pleasure. Liu et al. (2012) translated and adapted the scale into Chinese, confirming its reliability and validity. The Chinese version consists of 14 items rated on a four-point scale (1: strongly agree, 4: strongly disagree), with higher total scores indicating a more severe degree of anhedonia.

#### 2.2.4. The Chinese Version of the Temporal Experience of Pleasure Scale (TEPS)

The TEPS was developed by Gard et al. (2006) to assess anticipatory and consummatory pleasure experience. It was translated into Chinese by Chan et al. (2012a) and was shown to have good reliability. The Chinese version includes 20 items rated on a six-point Likert scale (1: not like me at all, 6: very much like me). In this study, the expected pleasure subscale was used to represent anticipatory pleasure, while the immediate pleasure subscale was used to assess consummatory pleasure.

#### 2.2.5. The Online Reward Motivation Adaptation Task (RMAT)

The Reward Motivation Adaptation Task (RMAT) in Study 1 is an online version of the same paradigm developed by Yan et al. (2024). The independent variable in this paradigm is the effort–reward relationship with three levels: effort–reward balance (Average, A), effort > reward imbalance (Bottom, B), and effort < reward imbalance (Top, T). The dependent variable is the reward motivation (‘wanting’ and ‘liking’). The entire paradigm consisted of eight blocks (ABATATAB) with 60 trials in total, 20 trials for each condition. Before the experiment, participants were asked to perform key press responses quickly and accurately. Before each trial, participants rated their ‘wanting’ (1: not at all, 7: very much). They then completed six “±8” mental arithmetic problems, with red numbers indicating addition and blue numbers indicating subtraction. If participants failed to respond to two of the questions, they had to repeat the mental arithmetic task for that trial. After each trial, the screen displayed the number of correctly answered questions, and the participants were asked to enter the expected reward value and rate the anticipatory pleasant experience (1: very happy, 7: very unhappy). Subsequently, actual reward values were given, and the effort–reward relationship was manipulated based on the difference between expected and actual rewards. Finally, participants rated their ‘liking’ (1: very happy, 7: very unhappy) by comparing the actual reward value with the expected reward value. For ease of interpretation, ‘liking’ ratings were reverse-coded such that higher scores indicate greater liking (i.e., more positive affect). Anticipatory pleasant-experience ratings were reverse-coded in the same way, with higher scores indicating greater anticipatory pleasure.

### 2.3. Procedures

College student participants were recruited through advertisements and posters. Participants completed an online questionnaire, which collected demographic information and included the PHQ-9, CSAS, SHAPS, and TEPS. For participants who met the inclusion criteria, they received a text message notification to complete the online RMAT behavioral task. Informed consent was obtained before the online questionnaire. The timing between questionnaire screening and completion of the online RMAT varied across participants; the median absolute interval was 33 days (interquartile range 17–179 days; range 0–227 days). After completing the study, participants were rewarded according to their performance in the RMAT. Ethical approval for this study was obtained from the Research Ethics Committee of the Institute of Psychology, Chinese Academy of Sciences (protocol number H22106).

### 2.4. Data Analysis

Data were initially collected from 192 participants. We applied a priori data-quality screening to ensure that the behavioral indices could be meaningfully interpreted. Of the 192 recruited participants, 134 provided complete behavioral data covering all 60 trials. After removing duplicate submissions from the same participant, 119 unique participants remained; when a participant completed the task more than once, we retained the most recent submission for analysis. Five additional participants were excluded because the first task block (used to index baseline ‘wanting’ at task onset) was missing, yielding a final analytic sample of 114 participants.

For each participant, mean ‘wanting’ and ‘liking’ ratings were extracted from each task block. Repeated-measures ANOVAs were conducted to examine the mean ‘wanting’ and ‘liking’ ratings across the three effort–reward conditions for the three screening-based groups (HSA, SD-noHSA, and HC). The interaction between group and effort–reward condition was treated as the primary test of adaptation in ‘wanting’ and ‘liking’ in Study 1. The family-wise error rate across these two primary tests was controlled using the Holm procedure with an overall alpha level of 0.05. To address concerns about statistical power for interaction effects, we conducted post hoc and sensitivity analyses in G*Power 3.1.9.7 (Faul et al., 2009) for the mixed-design repeated-measures ANOVA (within–between interaction; *α* = 0.05; three groups; three repeated measurements; correlation among repeated measures = 0.50; *ε* = 1.00). With the final analytic sample (N = 114), the achieved power was 0.520 for a small interaction effect (*f* = 0.10) and 0.999 for a medium effect (*f* = 0.25). Sensitivity analysis indicated that the minimum detectable interaction effect at 80% power was *f* = 0.134.

To capture individual differences in reward motivation adaptation, growth mixture modeling (GMM) was applied to the block-wise ‘wanting’ ratings to classify participants into high- and low-reward adaptation classes. This was specified a priori as an exploratory secondary analysis rather than a primary test of the study hypotheses. Given the sample size relative to the complexity of mixture models, the GMM analysis was conducted to describe potential heterogeneity in adaptation patterns and to generate hypotheses for future confirmatory work. Model selection was guided by standard fit indices and class interpretability criteria, with full details reported in the Supplementary Materials. Class differences in ‘liking’ across effort–reward conditions were then examined between adaptation classes using a repeated-measures ANOVA. Independent-samples *t*-tests compared questionnaire scores between reward motivation adaptation classes. Finally, a chi-square test was conducted to examine group distribution between the high and low reward motivation adaptation classes.

### 2.5. Results

#### 2.5.1. Demographic Information

Data from 192 participants were initially collected through an online behavioral task. After screening for incomplete trials, repeated submissions from the same participant, and missing baseline ‘wanting’ ratings from the first block, 114 participants’ data were retained for analysis. Table 1 summarizes the participant characteristics for the three screening-based groups (HSA, SD-noHSA, and HC).

#### 2.5.2. Repeated Measures ANOVA on Reward Motivation

**Figure 1:**
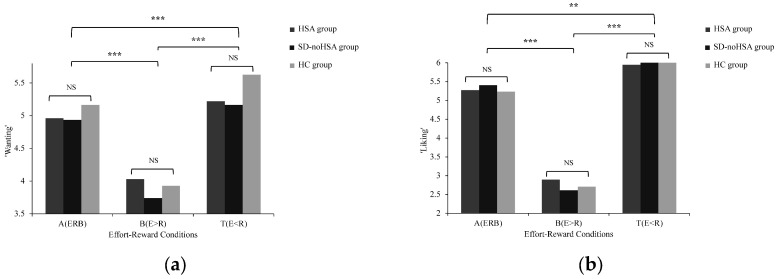
Reward motivation across effort–reward conditions in the HSA, SD-noHSA, and HC groups. (**a**) ‘wanting’ ratings; (**b**) ‘liking’ ratings (reverse-coded such that higher scores indicate greater liking). Ratings are shown as mean ± SEM. A = effort–reward balance; B = effort > reward imbalance; T = effort < reward imbalance. HSA = high social anhedonia group; SD-noHSA = subclinical depression without high social anhedonia group; HC = healthy control group. ** *p* < 0.01, *** *p* < 0.001; NS = not significant.

No significant Group × Condition interactions were found for either ‘wanting’ or ‘liking’ [‘wanting’: F(2.502, 138.860) = 1.145, *p* = 0.329, *η*^2^*_partial_* = 0.020; ‘liking’: F(1.322, 195.723) = 0.504, *p* = 0.731, *η*^2^*_partial_* = 0.010], indicating that the adaptation of reward motivation across effort–reward conditions did not differ reliably between the HSA, SD-noHSA, and HC groups. The main effect of groups on motivation was not significant [‘wanting’: F(2, 111) = 0.609, *p* = 0.546, *η*^2^*_partial_* = 0.011; ‘liking’: F(2, 148) = 0.050, *p* = 0.985, *η*^2^*_partial_* = 0.001], indicating the levels of reward motivation were similar across the three groups. The main effects of the effort–reward conditions were significant [‘wanting’: F(1.251, 138.860) = 110.295, *p* < 0.001, *η*^2^*_partial_* = 0.498; ‘liking’: F(1.322, 195.723) = 570.391, *p* < 0.001, *η*^2^*_partial_* = 0.794], indicating that all the participants adapted their reward motivation across both ‘wanting’ and ‘liking’ components. The descriptive results of reward motivation across effort–reward conditions and between-group comparisons are presented in Tables S1 and S2. Specifically, reward motivation was significantly higher in the effort–reward balance (A condition) compared to the effort > reward imbalance (B condition) and significantly lower than in the effort < reward imbalance (T condition). Additionally, reward motivation in the effort > reward imbalance (B condition) was significantly lower than in the effort < reward imbalance (T condition), as shown in Figure 1.

#### 2.5.3. GMM-Based Classification of Reward Motivation Adaptation

**Figure 2:**
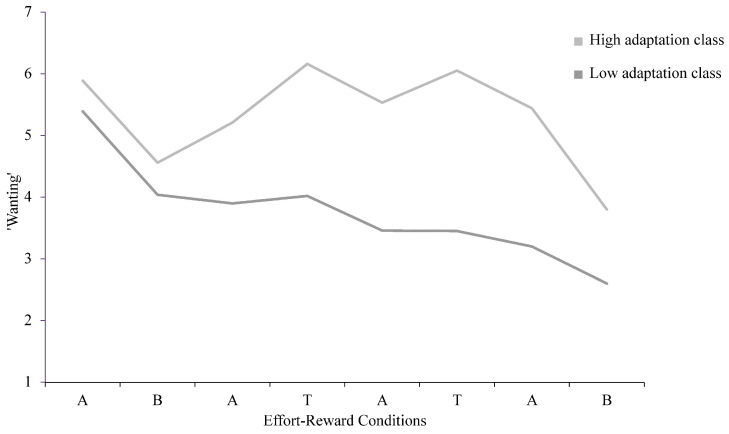
GMM-derived classes of reward motivation adaptation. Classes were identified using growth mixture modeling on block-wise ‘wanting’ ratings. The high-adaptation class showed a stronger adjustment of ‘wanting’ across effort–reward contexts than the low-adaptation class.

Following the group-level analyses, we conducted an exploratory GMM analysis on block-wise ‘wanting’ ratings to probe potential heterogeneity in reward motivation adaptation. Based on model fit and class interpretability (see Table S3 for model-selection details), a two-class solution was selected. This model classified 77 participants (67.5%) into a high-adaptation class and 37 participants (32.5%) into a low-adaptation class (Figure 2). Demographic characteristics did not differ substantially between the adaptation classes (see Table S4).

To validate the adaptation classification, we examined whether the two classes differed in ‘liking’ across effort–reward conditions. A repeated-measures ANOVA revealed a significant Class × Condition interaction, F(1.402, 157.027) = 18.549, *p* < 0.001, *η*^2^*_partial_* = 0.142. The main effect of condition was significant, F(1.402, 157.027) = 349.521, *p* < 0.001, *η*^2^*_partial_* = 0.757, and the main effect of class was also significant, F(1, 112) = 33.429, *p* < 0.001, *η*^2^*_partial_* = 0.230. Post hoc tests indicated higher ‘liking’ in the high-adaptation class than in the low-adaptation class in Condition A and Condition T, but not in Condition B (see Figure S1; Table S5). Together, these results suggest that the adaptation-based classification captured behaviorally coherent differences in reward experience (‘liking’) across effort–reward conditions.

To further evaluate the psychological relevance of the adaptation classes, we compared questionnaire measures between classes. Consistent with the role of anticipatory pleasure in effort expenditure, the high-adaptation class scored higher on the TEPS concrete anticipatory subscale than the low-adaptation class, *t*(95) = 3.020, *p* = 0.003, Cohen’s *d* = 0.664. This pattern supports the interpretation that the adaptation classes reflect individual differences relevant to anticipatory pleasure. In descriptive terms, the high-adaptation class showed stronger context-dependent modulation of ‘wanting’ and ‘liking’ accompanied by higher anticipatory pleasure, whereas the low-adaptation class reflects a flatter adaptation pattern with blunted anticipatory pleasure. Questionnaire comparisons are also reported in Table S4.

#### 2.5.4. Chi-Square Test Between Screening-Based Groups and Adaptation Classes

A chi-square test revealed no significant associations between adaptation class membership and the screening-based groups (HSA, SD-noHSA, and HC), *χ*^2^(2) = 3.921, *p* = 0.141 (see Table S6). This suggests that the adaptation-based classification was not simply a re-coding of the original screening-based group categorization.

### 2.6. Discussion

Study 1 demonstrated that all participants dynamically adjusted their reward motivation in response to the effort–reward ratio across both ‘wanting’ and ‘liking’ components. Contrary to our hypotheses, neither the HSA group nor the SD-noHSA group differed from the HC group in overall levels of reward motivation or in adaptation across conditions, and the subclinical groups were not overrepresented in the low-adaptation class. Thus, the primary analyses did not provide evidence for adaptation deficits in the predefined subclinical trait groups.

Several factors may account for the intact reward motivation adaptation in the HSA group and the SD-noHSA group. First, participants who completed the entire online experiment may have been subject to self-selection bias, resulting in a sample with relatively higher task engagement and reward motivation, which could attenuate between-group contrasts. Second, the interval between the initial trait screening and the behavioral task may have introduced classification noise, given that depressive symptoms assessed by the PHQ-9 can fluctuate over relatively short time windows, rendering the measure sensitive to temporal variation (Baryshnikov et al., 2023; Collins et al., 2024; Löwe et al., 2004). Consistent with this concern, the screening-to-task timing showed substantial variability in the present sample, with absolute intervals spanning 0 to 227 days. Collectively, the online recruitment procedures and variability in screening–task delays may have reduced the stability of the screening-based group classifications at the time of behavioral testing and contributed to the null group findings.

Importantly, exploratory person-level modeling suggested meaningful heterogeneity in reward-motivation adaptation. Because mixture-model solutions can be sensitive to sample size and model specification, these class findings are interpreted as descriptive and hypothesis-generating rather than confirmatory evidence. GMM analysis applied to block-wise ‘wanting’ delineated high- and low-adaptation classes characterized by distinct ‘liking’ responses across effort–reward conditions. Notably, the high-adaptation class exhibited higher anticipatory pleasure on the TEPS. This is consistent with prior evidence that anticipatory pleasure predicts willingness to expend effort for reward and is closely linked to motivational engagement (Geaney et al., 2015; Horne et al., 2021).

From a broader perspective, these results suggest that single-trait groupings based solely on social anhedonia or subclinical depression scores may not fully capture the multidimensional nature of reward-related functioning in subclinical populations. In naturalistic settings, subclinical depression often involves combinations of symptom dimensions, including anhedonia, that may jointly shape reward motivation (Serretti, 2023; Treadway & Zald, 2011). To address these design constraints and to reduce screening–task delays, Study 2 recruited a female subclinical depression sample characterized by additional anhedonia-related measures and used a supervised offline version of the RMAT to further examine whether reward motivation differences are more evident when depressive symptoms co-occur with elevated anhedonia.

## 3. Study 2

Considering that females have approximately twice the lifetime risk of developing depression compared to males, a disparity that emerges in mid-adolescence and persists into old age (Hankin et al., 2015; Luppa et al., 2012; Otte et al., 2016; Patton et al., 2008), Study 2 focused on female participants with subclinical depression and examined whether they exhibited deficits in reward motivation and its adaptation in terms of ‘wanting’ and ‘liking’. Study 2 extended Study 1 by testing reward motivation adaptation in an offline version of RMAT among female participants. Participants were assigned to a subclinical depression group (SD) and a healthy control group (HC) based on PHQ-9 screening. Anhedonia-related questionnaires were used to characterize pleasure- and motivation-related symptoms within this sample. We hypothesized that, in this female sample, participants in the SD group would show lower overall reward motivation and weaker adaptation across effort–reward conditions than HC participants.

### 3.1. Participants

A priori power analysis was conducted using G*Power 3.1.9.7 (Faul et al., 2009) for a mixed-design repeated-measures ANOVA (within–between interaction). Assuming a medium effect size (Cohen’s *f* = 0.25), *α* = 0.05, and power (1 − *β*) = 0.80, with two groups and three repeated measurements, the estimated minimum total sample size was 28 (with the default settings of correlation among repeated measures = 0.50 and nonsphericity correction *ε* = 1.00).

A total of 63 female college students were recruited from universities in Beijing, China, through online screening, and eligible students were invited via text message to participate in the behavioral experiment. Participants were assigned to one of two groups based on PHQ-9 screening: a subclinical depression group (SD) and a healthy control group (HC). The inclusion and exclusion criteria for the two groups are as follows: SD group: (1) PHQ-9 score greater than 10; (2) currently enrolled in a higher education institution; (3) female. HC group: (1) PHQ-9 score less than 5; (2) currently enrolled in a higher education institution; (3) female. Anhedonia-related questionnaires were administered to quantify pleasure and motivational symptoms at the symptom level, but were not used as inclusion criteria or to define group membership. The exclusion criteria for the two groups include: (1) taking psychotropic drugs; (2) diagnosis or history of other mental disorders; (3) history of substance dependence or abuse; (4) history of localized brain injury or neurological diseases.

### 3.2. Measures

In Study 2, the RMAT paradigm and the self-report measures (PHQ-9, SHAPS, and TEPS) were the same as in Study 1. The RMAT retained the same block structure (ABATATAB), number of trials per condition, and rating scales for ‘wanting’ and ‘liking’ as in Study 1, but was administered offline in the laboratory. The following additional questionnaires were administered:

#### 3.2.1. The Chinese Version of the Motivation and Pleasure Scale Self Report (MAP-SR)

The MAP-SR is a scale developed by Llerena et al. (2013) based on Clinical Assessment of Negative Symptoms (CAINS). L. Wang et al. (2020) translated the scale into Chinese and confirmed its reliability. The Chinese version includes four sections: social, recreational and work, interpersonal, and motivational, with a total of 15 items. It uses a five-point Likert scale (0: not at all/none, 4: very/extremely) to measure the degree and frequency of events, with lower scores indicating weaker reward motivation.

#### 3.2.2. The Chinese Version of the Effort–Reward Imbalance—School Version Questionnaire (ERI-S)

The ERI-S was developed by Li et al. (2010) as a translation and modification of the Effort–Reward Imbalance Scale developed by Siegrist et al. (2004). The scale has three dimensions: effort, reward, and overcommitment, with a total of 19 items rated on a five-point Likert scale (1: completely disagree, 5: completely agree). The effort–reward ratio is calculated to quantify the effort–reward imbalance.

#### 3.2.3. The Chinese Version of the Positive and Negative Affect Scale (PANAS)

The PANAS is a scale developed by Watson et al. (1988), translated into Chinese by Huang et al. (2003), and validated to measure recent mood states. The scale consists of 20 rating items measuring positive and negative emotions.

### 3.3. Procedures

Firstly, an online questionnaire was distributed in universities in Beijing, where participants completed the PHQ-9 scale online and provided basic demographic information. Participants who met the inclusion criteria for the SD group or the HC group were invited via text message to participate in the behavioral experiment. Compared with the online administration in Study 1, the offline laboratory setting in Study 2 allowed us to standardize instructions, monitor task engagement, and reduce environmental distractions during the RMAT. Furthermore, lab appointments were scheduled in close temporal proximity to the PHQ-9 screening wherever feasible, to reduce potential misclassification caused by fluctuations in depressive status between screening and task performance. During the lab session, participants first completed the RMAT, followed by the MAP-SR, TEPS, ERI-S, SHAPS, and PANAS, and then provided demographic information and signed informed consent for the behavioral experiment. After completing the experiment, participants were rewarded according to their performance in the RMAT. Ethical approval for this study was obtained from the Research Ethics Committee of the Institute of Psychology, Chinese Academy of Sciences (protocol number H22106).

### 3.4. Data Analysis

Data were originally collected from 63 participants. The final analysis included 60 participants after excluding participants with invariant ‘wanting’ ratings across all trials, which we treated as indicating non-adherence to task instructions because such flat profiles do not provide interpretable information about within-person adaptation.

For each participant, mean ‘wanting’ and ‘liking’ ratings were computed for each effort–reward condition and analyzed using mixed-design repeated-measures ANOVAs with condition (A, B, T) as the within-subject factor and group (SD vs. HC) as the between-subject factor. Reward motivation was indexed by two components, wanting and liking. We therefore treated the Group × Condition interactions for ‘wanting’ and for ‘liking’ as the two primary tests and controlled the family-wise error rate across these tests using the Holm–Bonferroni correction with an alpha level of 0.05. To characterize the detectable effect sizes given the final sample size, we conducted post hoc and sensitivity analyses for the within–between interaction in the mixed-design repeated-measures ANOVA using G*Power 3.1.9.7 (Faul et al., 2009; *α* = 0.05; two groups; three repeated measurements; correlation among repeated measures = 0.50; nonsphericity correction *ε* = 1.00). With the final analytic sample (N = 60), achieved power was 0.370 for a small interaction effect (*f* = 0.10) and 0.991 for a medium effect (*f* = 0.25). Sensitivity analysis indicated that the minimum detectable interaction effect at 80% power was *f* = 0.166.

Subsequently, a regression analysis was employed to quantify reward motivation adaptation. The regression coefficient of the effort–reward ratio was calculated for each participant, controlling for trial number, and served as the quantitative adaptation index. Finally, independent samples *t*-tests were conducted to compare this index and other psychometric scale scores between groups.

### 3.5. Results

#### 3.5.1. Demographic Characteristics and Questionnaire Scores

After excluding those individuals who provided identical ratings across all conditions, data from 60 participants were included in the final analysis, with 30 in the subclinical depression group (SD) and 30 in the healthy control group (HC). Table 2 presents demographic characteristics and baseline questionnaire scores. The two groups did not differ significantly in age, years of education, or parental age and education (all *ps* > 0.05). In contrast, compared with the HC group, the SD group reported lower pleasure experience and motivation on the MAP-SR, lower abstract anticipatory pleasure on the TEPS, a higher effort–reward ratio on the ERI-S, higher anhedonia on the SHAPS (including both total and dichotomous scores), and a more negative affective profile on the PANAS (lower positive affect and higher negative affect; Table 2). Thus, although group membership was defined by PHQ-9 scores, the SD group also showed higher anhedonia and greater effort–reward imbalance burden than the HC group at baseline.

#### 3.5.2. Repeated Measures ANOVA on Reward Motivation

**Figure 3:**
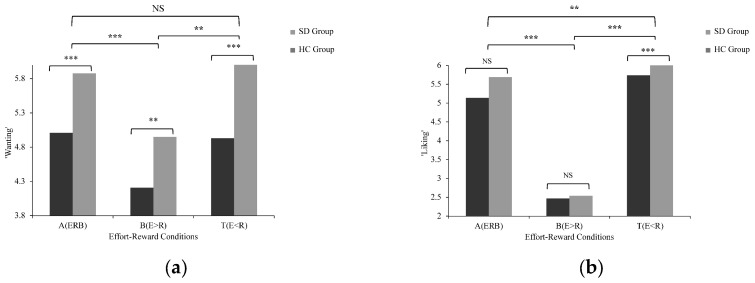
Comparison of reward motivation ratings between the subclinical depression (SD) group and the healthy control (HC) group across effort–reward conditions: (**a**) ‘wanting’; (**b**) ‘liking’ (reverse-coded such that higher scores indicate greater liking). Values are shown as mean ± SEM. A = effort–reward balance; B = effort > reward imbalance; T = effort < reward imbalance. ** *p* < 0.01, *** *p* < 0.001; NS = not significant.

A repeated-measures ANOVA with effort–reward condition (A, B, T) as a within-subject factor and Group (SD vs. HC) as a between-subject factor was conducted for ‘wanting’ and ‘liking’. Because reward motivation was indexed by two components, we treated the Group × Condition interactions for ‘wanting’ and ‘liking’ as the two primary tests in Study 2 and controlled the family-wise error rate across these tests using the Holm procedure (overall α = 0.05). For ‘wanting’, there was no significant interaction between group and effort–reward condition [F(1.273, 73.818) = 1.423, *p* = 0.243, *η*^2^*_partial_* = 0.024], indicating that the SD and HC groups showed similar adaptation for ‘wanting’ across effort–reward conditions. For ‘liking’, there was a nominally significant interaction between group and effort–reward condition [F(1.293, 75.007) = 3.897, *p* = 0.042, *η*^2^*_partial_* = 0.063], but it did not survive the Holm-adjusted significance threshold, so evidence for group differences in ‘liking’ adaptation should be regarded as tentative. The main effect of the effort–reward condition was significant for reward motivation [‘wanting’: F(1.273, 73.818) = 41.979, *p* < 0.001, *η*^2^*_partial_* = 0.420; ‘liking’: F(1.293, 75.007) = 391.793, *p* < 0.001, *η*^2^*_partial_* = 0.871], indicating that ratings differed across effort–reward conditions. The results were consistent for both ‘wanting’ and ‘liking’ in the effort–reward balance (A condition) and the effort < reward imbalance (T condition). In the B condition, ‘wanting’ ratings for the SD group were still significantly lower than those of the HC group, but for ‘liking’, no significant difference was found between the two groups. The main effect of group was significant [‘wanting’: F(1, 58) = 20.269, *p* < 0.001, *η*^2^*_partial_* = 0.259; ‘liking’: F(1, 58) = 10.364, *p* = 0.002, *η*^2^*_partial_* = 0.152], with the SD group showing lower overall motivation than the HC group. Follow-up comparisons showed that the SD group reported lower ‘wanting’ than the HC group in all three conditions (A: F(1, 58) = 20.146, *p* < 0.001; B: F(1, 58) = 8.522, *p* = 0.005; T: F(1, 58) = 17.866, *p* < 0.001). For ‘liking’, the SD group was lower than the HC group in conditions A and T (A: F(1, 58) = 8.939, *p* = 0.004; T: F(1, 58) = 19.860, *p* < 0.001), but not in condition B (F(1, 58) = 0.071, *p* = 0.790), as shown in Figure 3 and Table S7.

#### 3.5.3. Differences in Reward Motivation Adaptation Indicators in SD and HC Groups

Separate regression equations were established with each participant’s ‘wanting’ and ‘liking’ rating across trials as the dependent variable, the effort–reward ratio (expected rewards/actual rewards) as the independent variable, and the number of trials as the control variable. The regression equations established for each of the dependent variables were as follows:

‘wanting’ = *β*_0_ + *β*_1,‘wanting’_ × effort-reward ratio + *β*_2_ × number of trials, (1)

‘liking’ = *β*_0_ + *β*_1,‘liking’_ × effort-reward ratio + *β*_2_ × number of trials. (2)

The regression coefficients *β*_1_ for ‘wanting’ and ‘liking’ quantified the reward motivation adaptation for each participant after controlling for the number of trials. We compared *β*_1_ for ‘wanting’ and ‘liking’ between the SD and HC groups. As shown in Table S8, an independent samples *t*-test found that the *β*_1_ coefficients in the SD group were lower than those in the HC group, and the difference for ‘liking’ approached borderline significance (‘wanting’: *t*(58) = −1.659, *p* = 0.103, Cohen’s *d* = −0.428; ‘liking’: *t*(58) = −1.948, *p* = 0.056, Cohen’s *d* = −0.503), which was directionally consistent with the ANOVA pattern but did not provide definitive evidence for group differences in adaptation slopes.

For other scale scores, Pearson correlation analyses were conducted across the full sample (N = 60) to examine associations between adaptation indices (*β*_1_ coefficients) and self-report measures. The correlation analysis revealed a strong positive correlation between *β*_1,‘wanting’_ and TEPS abstract_anticipatory (*r*(58) = 0.341, *p* = 0.008). There was also a significant positive correlation between *β*_1,‘liking’_ and the MAP relationship subscale (*r*(58) = 0.285, *p* = 0.028), and a significant negative correlation with the SHAPS dichotomous score (*r*(58) = −0.274, *p* = 0.034). Therefore, *β*_1,‘wanting’_ was positively correlated with abstract anticipatory pleasure, and *β*_1,‘liking’_ was positively correlated with relational reward motivation and negatively correlated with anhedonia. Together, these associations provide convergent evidence for the construct validity of the *β*_1_ indices as individual-difference markers of reward motivation adaptation.

### 3.6. Discussion

In this offline female sample, the SD group showed higher reported anhedonia on questionnaires assessing pleasure and motivation than the HC group, along with reduced reward motivation in the behavioral task. With respect to adaptation, the deficits of reward motivation adaptation in the SD group should be interpreted cautiously since the nominally significant interaction between Group and Condition for ‘liking’ did not survive family-wise error correction. No comparable interaction effect was observed for ‘wanting’. The adaptation indices (*β*_1,‘wanting’_ and *β*_1,‘liking’_) showed meaningful associations with measures of pleasure experience and anhedonia.

In contrast to Study 1, which focused on isolated subclinical trait groups (where the SD-noHSA group excluded high social anhedonia), Study 2 specifically examined a subclinical depression group whose symptom profile was characterized using broader measures of anhedonia. In this study, group membership was defined solely on the basis of PHQ-9 scores, and anhedonia was assessed with the SHAPS, MAP-SR, and related scales to characterize symptom burden rather than to determine inclusion. Accordingly, the SD group can be viewed as representing the combined presence of elevated subclinical depressive symptoms and higher anhedonia burden, rather than depressive symptoms in isolation. This approach aligns with previous research showing that anhedonia is prevalent in both clinical and subclinical depression (Treadway & Zald, 2011; Serretti, 2023) and is closely linked to reward motivation (Sherdell et al., 2012). Within this framework, the group differences observed in Study 2 are best interpreted as reflecting the combined presence of subclinical depressive status and elevated anhedonia, rather than the effect of depressive status in isolation. The results revealed diminished reward motivation in this group. The overall pattern tentatively suggested that potential adaptation differences, if present, might be more detectable in ‘liking’ than in ‘wanting’, which aligns with established theories positing ‘wanting’ and ‘liking’ as distinct components of reward motivation (Berridge, 2004; Berridge & Robinson, 2016). However, given the modest sample size and the sensitivity of the ‘liking’ interaction to multiple-testing control, this component-specific interpretation remains preliminary and requires confirmation in larger, preregistered studies.

## 4. General Discussion

Taken together, the present findings provide a mixed pattern of evidence for our initial hypotheses about reward motivation adaptation in subclinical depression. In the female sample, co-occurring subclinical depression and elevated anhedonia were associated with diminished reward motivation and potentially impaired ‘liking’ adaptation, based on post hoc analyses of anhedonia-related questionnaire scores. This pattern is compatible with recent work linking anhedonia burden to reward-related motivational impairments in depression-spectrum samples (Barkus, 2021; Hallford & Austin, 2022; Treadway et al., 2012; S. Wang et al., 2021).

One plausible account is that reward motivation impairments become most evident when anhedonia and depressive symptoms co-occur, reflecting their joint influence on both reward processing and the translation of reward value into motivated action. First, anhedonia is intrinsically linked to disruptions in reward-related processes, including reward valuation, reward learning, and hedonic capacity. Consistent with this view, elevated anhedonia has been associated with diminished willingness to expend effort for reward and with impairments in reward learning (Frey et al., 2023; Treadway et al., 2009; Vrieze et al., 2013), and converging evidence also implicates alterations in reward circuitry in anhedonia-related dysfunction (Borsini et al., 2020; Frey et al., 2023; Steinmann et al., 2022). Second, depressive symptoms beyond anhedonia may also contribute to motivational impairment, but their impact is not necessarily uniform across individuals. Grahek et al. (2019) emphasized the heterogeneity of depressive symptom profiles and proposed that different symptom dimensions may disrupt different links along the pathway from reward value to goal-directed behavior. In line with this framework, the association between depression and effort expenditure is not always consistent, yet becomes more detectable when anhedonia is considered jointly with depressive symptoms (Horne et al., 2021). Within this context, our data are compatible with the possibility that reward disruptions associated with anhedonia and motivational constraints related to depression jointly impair reward motivation adaptation; however, the present design does not allow this mechanism to be tested directly, and the pattern should therefore be regarded as hypothesis-generating rather than confirmatory. At the same time, the largely null interaction effects, combined with the post hoc power estimates, offer only weak grounds for drawing strong negative conclusions about reward motivation adaptation in subclinical depression.

One possible reason why adaptation differences appeared more clearly for ‘liking’ than for ‘wanting’ is that these components map onto partially distinct stages of reward processing and show different sensitivity to depressive symptoms. Meta-analytic evidence suggests that depression related abnormalities are more consistently observed in reward feedback and learning, whereas anticipation-related effects tend to be smaller and more variable across studies (Halahakoon et al., 2020; Keren et al., 2018; Whitton et al., 2015). Because ‘liking’ more directly reflects consummatory affective responses to reward outcomes, *β*_1,‘liking’_ may be more tightly coupled to feedback-related reward processing and therefore more sensitive to anhedonia-linked dysfunction. This interpretation is supported by the correlational pattern: *β*_1,‘liking’_ was negatively associated with anhedonia and positively associated with relational reward motivation, whereas *β*_1,‘wanting’_ was associated with abstract anticipatory pleasure. Taken together, these associations suggest that the adaptation index derived from ‘liking’ may preferentially capture individual differences in anhedonia-related reward responsiveness, which could help explain why adaptation differences were more apparent in *β*_1,‘liking’_ than in *β*_1,‘wanting’_ in the present female SD sample. However, these component-specific interpretations should be regarded as preliminary, particularly because the adaptation indices and the findings from the growth mixture model in Study 1 were exploratory, depended on complex modeling choices, and were based on modest sample sizes. Accordingly, we regard the group-level effects on ‘wanting’ and ‘liking’ as the primary confirmatory results of the present work, whereas the patterns involving anhedonia and the *β*_1_ indices, as well as the latent adaptation classes, are treated as exploratory signals that warrant replication rather than as definitive evidence for distinct motivational subtypes.

This study has several limitations. First, as it utilized behavioral experiments, it did not explore the corresponding neural mechanisms. Further research might delve into the cognitive and neural mechanisms underlying reward motivation adaptation, for example, combining effort–reward paradigms with neuroimaging markers of reward learning and prediction error (Halahakoon et al., 2020; Keren et al., 2018; Whitton et al., 2015) to clarify how adaptation relates to established reward-circuit alterations in depression and anhedonia. Recent evidence suggests that subthreshold depression already shows shared and distinct alterations in brain connectivity and cognitive function relative to MDD, highlighting the value of integrating behavioral adaptation indices with neural markers in future work (Zhong et al., 2026). Second, the effort–reward imbalance paradigm used in this study focused primarily on monetary rewards. Social rewards, however, play a significant role in daily life, and whether individuals with subclinical depression experience similar declines in reward motivation and adaptation within social reward contexts remains an open question for further investigation. Future studies could incorporate social rewards to explore how reward motivation and adaptation manifest within social contexts, extending recent work on social reward processing and anhedonia (Solomonov et al., 2023) and shedding light on the everyday social experiences of individuals with subclinical depression. Third, the online task recruitment with possible self-selection of participants may have attenuated group differences in Study 1. Thus, Study 2 was conducted. But the different recruitments and task administration may limit the direct comparability of the two studies. Fourth, Study 2 included only women, which reduces gender-related heterogeneity but also restricts the generalizability of its findings to men. Future work should examine whether similar patterns of reward motivation and adaptation are observed in mixed-sex samples. Fifth, we excluded participants with exactly the same ‘wanting’ ratings across trials, as this pattern likely reflects poor task adherence and precludes meaningful estimation of within-person changes. However, we acknowledge that such flat profiles could, in some cases, reflect severe apathy or anhedonia. Consequently, individuals with extremely blunted reward motivation may be underrepresented here, and future studies should specifically recruit and characterize this subgroup using additional behavioral and clinical measures. Finally, both studies focused on unmedicated subclinical samples rather than formally diagnosed clinical populations, and both designs were cross-sectional. Any potential clinical implications should be regarded as provisional until they are further evaluated in longitudinal and intervention studies.

Notwithstanding the limitations noted above, by integrating online and offline behavioral data, this study suggests that female participants with co-occurring subclinical depression and anhedonia may exhibit diminished reward motivation and adaptation specifically in the ‘liking’ domain under varying effort–reward ratios. It adds to the broader literature on reward processing in depression by applying an effort–reward imbalance paradigm to a subclinical female sample and by introducing quantitative indices of reward-motivation adaptation that can be taken forward in larger, preregistered clinical and longitudinal studies.

## 5. Conclusions

In conclusion, the present studies reveal a nuanced pattern of reward motivation in subclinical depression. Across online and offline samples, individuals with subclinical depression and elevated anhedonia consistently reported lower overall reward motivation. Context-dependent adaptation effects were modest, emerging only as small, liking-related differences in the female sample. Together, these findings suggest that adaptation failure with effort–reward ratio may reflect motivational dysfunction in depression. Detecting when and for whom such deficits arise will require larger, preregistered longitudinal and clinical studies with sufficient power to assess subtle context-dependent effects and directly test underlying mechanisms.

## Figures and Tables

**Table 1:** Demographic characteristics and task performance across groups in Study 1.

Variable	HSA (N = 38)		SD-noHSA (N = 38)		HC (N = 38)				
	Mean	SD	Mean	SD	Mean	SD	F/*χ*^2^	*df*	*p*
Age (years)	22.25	2.51	21.51	1.89	22.10	2.54	1.081	111	0.343
Gender (male/female)	16/22		14/24		13/25		0.523	2	0.770
Years of education (years)	14.66	1.86	14.05 ^1^	1.94	14.26 ^2^	2.01	0.939	108	0.394
Father’s years of education (years)	10.61	4.25	9.84 ^1^	3.18	11.03 ^2^	4.00	0.905	108	0.408
Mother’s years of education (years)	11.00	5.32	8.62 ^1^	3.53	10.75 ^2^	4.59	3.127	108	0.048
Calculation accuracy in RMAT	0.70	0.14	0.70	0.11	0.67	0.13	0.444	111	0.643

Note. Data are presented as mean (SD) unless otherwise indicated. Gender is presented as counts (male/female) and was analyzed using a Chi Square (χ^2^) test. Continuous variables were compared using one-way ANOVA. Parentheses indicate the sample size for variables with missing data. RMAT calculation accuracy is defined as the proportion of correct arithmetic solutions. HSA = high levels of social anhedonia group; SD-noHSA = subclinical depression without high levels of social anhedonia group; HC = the control group with low levels of both depression and social anhedonia. RMAT = Reward Motivation Adaptation Task. ^1^ N = 37 (SD-noHSA). ^2^ N = 36 (HC).

**Table 2:** Demographic characteristics and questionnaire scores in SD and HC groups in Study 2.

Variables		SD (N = 30)		HC (N = 30)					
		Mean	SD	Mean	SD	*t*	*df*	*p*	Cohen’s *d*
Demographic Characteristics									
Age (years)		21.30	3.08	21.47	2.83	−0.219	58	0.828	−0.057
Years of Education (years)		14.30	1.78	15.03	1.85	−1.564	58	0.123	−0.404
Father’s Age (years)		49.83	10.60	51.60	5.22	−0.819	58	0.416	−0.211
Father’s Education (years)		13.00	3.95	14.53	3.51	−1.590	58	0.117	−0.411
Mother’s Age (years)		49.20	4.63	48.93	4.53	0.225	58	0.822	0.058
Mother’s Education (years)		13.03	4.09	13.93	4.02	−0.860	58	0.393	−0.222
**Scale**	**Subscale**								
MAP-SR	Total	49.10	8.76	58.30	6.72	**−4.563**	**58**	**<0.001**	**−1.178**
TEPS	Abstract_anticipatory	18.27	3.67	20.23	2.91	**−2.302**	**58**	**0.025**	**−0.594**
	Concrete_anticipatory	17.73	4.22	16.83	4.37	0.812	58	0.420	0.210
	Abstract_consumatory	27.47	4.25	28.27	4.76	−0.687	58	0.495	−0.177
	Concrete_consumatory	18.17	3.29	17.00	3.53	1.323	58	0.191	0.342
	Total	85.67	13.42	87.13	12.26	0.196	58	0.660	−0.114
ERI-S	Effort–Reward ratio	1.14	0.38	0.87	0.24	**3.293**	**58**	**0.002**	**0.850**
SHAPS	Total	25.07	6.13	20.50	4.72	**3.235**	**58**	**0.002**	**0.835**
	Dichotomous Score	1.90	2.68	0.33	0.80	**3.065**	**34.146**	**0.004**	**0.791**
PANAS	Positive	22.83	4.80	30.87	6.95	**−5.211**	**51.551**	**<0.001**	**−1.345**
	Negative	26.17	7.65	17.23	4.44	**5.531**	**46.530**	**<0.001**	**1.428**

Note. Data are presented as mean (SD). Group comparisons were conducted using independent-samples *t*-tests; Welch-adjusted degrees of freedom are reported when variances were unequal. Cohen’s d is reported as (SD − HC), such that negative values indicate lower scores in the SD group. SD = subclinical depression group; HC = healthy control group. MAP-SR = Motivation and Pleasure Scale—Self Report; TEPS = Temporal Experience of Pleasure Scale; ERI-S = Effort–Reward Imbalance—School Version; SHAPS = Snaith–Hamilton Pleasure Scale; PANAS = Positive and Negative Affect Schedule. Bold values indicate statistically significant group differences at *p* < 0.05.

## Data Availability

The data that support the findings of this study are available from the corresponding author upon reasonable request.

## Supplementary Materials

The following supporting information can be downloaded at: https://www.mdpi.com/article/10.3390/bs16030464/s1. Table S1: ‘Wanting’ across effort–reward conditions and screening-based group comparisons; Table S2: ‘Liking’ across effort–reward conditions and screening-based group comparisons; Table S3: Model fit indices for growth mixture models of reward-motivation adaptation; Table S4: Demographic information and trait scale scores of high- and low-adaptation classes; Table S5: Comparison of ‘liking’ between high- and low-adaptation classes across effort–reward conditions; Table S6: Counts and percentages of high and low adaptation by screening-based group; Table S7: Repeated-measures ANOVA results for ‘wanting’ and ‘liking’ across effort–reward conditions; Table S8: Comparison of reward-motivation indicators (*β*_1_) for ‘wanting’ and ‘liking’ between SD and HC groups; Figure S1: Behavioral validation of high- and low-adaptation classes based on ‘liking’. (Franken et al., 2007; Snaith et al., 1995) are cite in Supplementary Materials.
