# Supplementary material for: Reward Motivation Adaptation Deficits Are Specific to Co-Occurring Subclinical Depression and Anhedonia
Source: Behav Sci (Basel). 2026 Mar 20;16(3):464. doi: 10.3390/bs16030464 (PMC13024454; doi:10.3390/bs16030464)
Supplement: Supplementary file 1 [file behavsci-16-00464-s001.zip › behavsci-4100747-supplementary.docx]

**Supplementary Materials**

This Supplementary Materials includes: descriptive statistics for Study 1 (Table S1 –S2), GMM model selection (Table S3), questionnaire comparisons for adaptation classes (Table S4), validation analyses (Table S5 & Figure S1), trait × class counts (Table S6), and Study 2 follow-up and *β*_1_ indices (Tables S7 – S8).

**Table S1** ‘Wanting’ Across Effort-Reward Conditions and Screening-Based Group Comparisons

|  | Effort-Reward Balance(A) | Effort>  Reward Imbalance(B) | Effort<  Reward Imbalance (T) | A-B | | A-T | | B-T | |
| --- | --- | --- | --- | --- | --- | --- | --- | --- | --- |
|  |  |  |  | Difference  (95% CI) | *p* | Difference  (95% CI) | *p* | Difference  (95% CI) | *p* |
| HSA  (N=38)  Mean (SD) | 4.96(0.18) | 4.03(0.23) | 5.22(0.23) | 0.932  (0.541~1.323) | <.001 | -0.260  (-0.540~0.020) | .078 | -1.192  (-1.757~-0.628) | <.001 |
| SD-noHSA  (N=38)  Mean (SD) | 5.17(0.18) | 3.93(0.23) | 5.63(0.23) | 1.235  (0.844~1.627) | <.001 | -0.461  (-0.741~-0.181) | <.001 | -1.696  (-2.261~-1.132) | <.001 |
| HC  (N=38)  Mean (SD) | 4.94(0.18) | 3.74(0.23) | 5.17(0.23) | 1.196  (0.804~1.587) | <.001 | -0.229  (-0.509~0.051) | .147 | -1.425  (-1.989~-0.861) | <.001 |

**Note.** Values are mean (SE/SD; use the one consistent with the table) for each trait group. A = effort–reward balance; B = effort > reward imbalance; T = effort < reward imbalance. Pairwise contrasts (A–B, A–T, B–T) are computed as the first condition minus the second condition; positive values indicate higher ratings in the first condition. 95% confidence intervals are reported for the mean difference. HSA = high social anhedonia group; SD-noHSA = subclinical depression without high social anhedonia group; HC = healthy control group.

**Table S2** ‘Liking’ Across Effort-Reward Conditions and Screening-Based Group Comparisons

|  | Effort-Reward Balance (A) | Effort>  Reward Imbalance (B) | Effort<  Reward Imbalance (T) | A-B | | A-T | | B-T | |
| --- | --- | --- | --- | --- | --- | --- | --- | --- | --- |
|  |  |  |  | Difference  (95% CI) | *p* | Difference  (95% CI) | *p* | Difference  (95% CI) | *p* |
| HSA  (N=38)  Mean (SD) | 5.28(0.14) | 2.90(0.18) | 5.95(0.16) | 2.382  (1.842~2.922) | <.001 | -0.670  (-0.972~-0.367) | <.001 | -3.051  (-3.687~-2.416) | <.001 |
| SD-noHSA  (N=38)  Mean (SD) | 5.24(0.14) | 2.71(0.18) | 6.06(0.16) | 2.530  (1.990~3.069) | <.001 | -0.826  (-1.129~-0.524) | <.001 | -3.356  (-3.991~-2.721) | <.001 |
| HC  (N=38)  Mean (SD) | 5.41(0.14) | 2.61(0.18) | 6.01(0.16) | 2.796  (2.256~3.336) | <.001 | -0.599  (-0.901~-0.296) | <.001 | -3.395  (-4.030~-2.759) | <.001 |

**Note.** Values are mean (SE/SD; use the one consistent with the table) for each trait group. ‘Liking’ ratings are reverse-coded such that higher values indicate greater liking. A = effort–reward balance; B = effort > reward imbalance; T = effort < reward imbalance. Pairwise contrasts (A–B, A–T, B–T) are computed as the first condition minus the second condition; positive values indicate higher ratings in the first condition. 95% confidence intervals are reported for the mean difference. HSA = high social anhedonia group; SD-noHSA = subclinical depression without high social anhedonia group; HC = healthy control group.

**Table S3** Model Fit Indices for Growth Mixture Models of Reward Motivation Adaptation

| Model | | k | G^2/LL | AIC | BIC | aBIC | Entropy | LMR (p value) | BLRT (p value) | Class probabilities |
| --- | --- | --- | --- | --- | --- | --- | --- | --- | --- | --- |
| LCGM | 2C | 18 | -1455.793 | 2947.586 | 2996.837 | 2939.945 | 0.946 | 0.0008 | <0.001 | .535/.465 |
|  | 3C | 21 | -1408.635 | 2859.270 | 2916.730 | 2850.356 | 0.920 | 0.1850 | <0.001 | .465/.254/.281 |
|  | 4C | 24 | -1385.203 | 2818.405 | 2884.074 | 2808.218 | 0.899 | 0.0787 | <0.001 | .140/.255/.263/.342 |
| GMM | 2C | 21 | -1368.511 | 2779.022 | 3743.081 | 2770.109 | 0.890 | 0.0019 | <0.001 | .702/.298 |
|  | 3C | 24 | -1353.224 | 2754.449 | 2836.482 | 2744.262 | 0.851 | 0.1069 | <0.001 | .316/.386/.298 |
|  | 4C | 27 | -1368.511 | 2791.022 | 2864.900 | 2779.562 | 0.945 | 0.5000 | 1.0000 | .298/0/0/.702 |
| **GMM-free** | **2C** | **29** | **-1290.874** | **2639.747** | **2719.097** | **2627.438** | **0.917** | **0.4049** | <0.001 | **.675/.325** |
|  | 3C | 40 | -1186.426 | 2452.853 | 2562.301 | 2435.874 | 0.976 | 0.4991 | 1.0000 | .895/.105/0 |
|  | 4C | 43 | -1251.581 | 2589.162 | 2706.819 | 2570.910 | 0.930 | 0.6333 | <0.001 | .674/.202/0/.123 |

**Note.** Latent class growth models (LCGM), conventional growth mixture models (GMM), and variance–covariance–unconstrained growth mixture models (GMM-free) were fitted to mean “wanting” ratings across RMAT task blocks to identify subgroups differing in reward motivation adaptation. k = number of free parameters; G²/LL = log-likelihood–based fit statistic; AIC = Akaike information criterion; BIC = Bayesian information criterion; aBIC = sample-size–adjusted BIC; higher entropy indicates greater classification precision. LMR = Lo–Mendell–Rubin likelihood ratio test; BLRT = bootstrap likelihood ratio test. Class probabilities indicate the estimated proportion of participants in each latent class for a given solution. The two-class GMM-free solution was retained as the final model reported in the main text.

**Table S4** Demographic Information and Trait Scale Scores of High and Low Adaptation Classes

|  | | High adaptation class  (N=77) | | Low adaptation class  (N=37) | |  |  |  |  |
| --- | --- | --- | --- | --- | --- | --- | --- | --- | --- |
|  | | Mean | SD | Mean | SD | t /χ2 | *df* | *p* | Cohen’s *d* |
| Age (years) | | 21.86 | 2.26 | 22.15 | 2.50 | -.612 | 112 | .542 | -.122 |
| Sex (M/F) | | 27/50 | | 16/21 | | .712 | 1 | .399 |  |
| Years of education (years) | | 14.32  (N=74) | 1.76 | 14.35 | 2.28 | -.086 | 109 | .931 | -.017 |
| Father's education (years) | | 10.14  (N=74) | 3.49 | 11.19 | 4.41 | -1.371 | 109 | .173 | -.276 |
| Mother's years of education (years) | | 9.43  (N=74) | 4.37 | 11.51 | 4.76 | -2.296 | 109 | .024 | -.462 |
| Calculation accuracy | | 0.69 | 0.13 | 0.68 | 0.12 | .472 | 112 | .638 | .094 |
| PHQ | | 7.84 | 5.50 | 6.70 | 4.48 | 1.099 | 112 | .274 | .220 |
| CSAS | | 13.66 | 8.39 | 14.76 | 8.82 | -.642 | 112 | .522 | -.128 |
| SHAPS | Total | (N=67)  23.15 | (N=67)  7.29 | (N=30)  21.47 | (N=30)  5.30 | 1.135 | 95 | .259 | .249 |
|  | Dichotomous Score | (N=67)  1.34 | (N=67)  2.96 | (N=30)  0.37 | (N=30)  0.72 | **2.541** | **81.33** | **.013** | **.317** |
| TEPS | Abstract_anticipatory | (N=67)  20.00 | (N=67)  3.10 | (N=30)  20.07 | (N=30)  3.69 | -.092 | 95 | .927 | -.020 |
|  | Concrete_anticipatory | (N=67)  19.22 | (N=67)  4.88 | (N=30)  16.03 | (N=30)  4.65 | **3.020** | **95** | **.003** | **.664** |
|  | Abstract_consumatory | (N=67)  28.00 | (N=67)  4.56 | (N=30)  28.90 | (N=30)  4.84 | -.882 | 95 | .380 | -.194 |
|  | Concrete_consumatory | (N=67)  18.24 | (N=67)  3.63 | (N=30)  19.00 | (N=30)  2.79 | -1.021 | 95 | .310 | -.224 |
|  | Total | (N=67)  89.69 | (N=67)  13.92 | (N=30)  88.47 | (N=30)  10.64 | .427 | 95 | .670 | .094 |

**Note.** Values are mean (SD) unless otherwise indicated. Sex is reported as counts (M/F) and tested using *χ²*. Continuous variables were compared using independent-samples *t* tests; Welch-adjusted degrees of freedom are reported when variances were unequal. Cohen’s *d* is computed as (High adaptation − Low adaptation), such that positive values indicate higher scores in the high-adaptation group. PHQ-9 = Patient Health Questionnaire-9; CSAS = Chapman Social Anhedonia Scale; SHAPS = Snaith–Hamilton Pleasure Scale; TEPS = Temporal Experience of Pleasure Scale. SHAPS dichotomous means in both groups were below the commonly used screening cutoff (>2) for anhedonia (Franken et al., 2007; Snaith et al., 1995); thus, the SHAPS between-class difference should be interpreted cautiously as an exploratory finding.

**Table S5** Comparison of ‘Liking’ Between High and Low Adaptation Classes Across Effort-Reward Conditions

| Condition | High Adaptation class  (N=77)  Mean (SD) | Low Adaptation class  (N=37)  Mean (SD) | F(1,112) | *p* |
| --- | --- | --- | --- | --- |
| Effort-reward balance (A) | 5.57(0.84) | 4.74(0.58) | 29.280 | <.001 |
| Effort>reward imbalance (B) | 2.70(1.18) | 2.82(1.00) | .313 | .577 |
| Effort<reward imbalance (T) | 6.43(0.55) | 5.12(1.12) | 70.593 | <.001 |

**Note.** Values are mean (SD). ‘Liking’ ratings are reverse-coded such that higher values indicate greater liking. A = effort–reward balance; B = effort > reward imbalance; T = effort < reward imbalance. Reported F(1,112) and p values test between-class differences (high vs. low adaptation) within each condition.

**Table S6** Counts and Percentages of High and Low Adaptation by Screening-Based Group

|  | | | Adaptation class | | |
| --- | --- | --- | --- | --- | --- |
|  | | | High | Low | Total |
| Trait Group | HSA | Count | 22 | 16 | 38 |
|  |  | Percentage | 57.9% | 42.1% | 100% |
|  | SD-noHSA | Count | 30 | 8 | 38 |
|  |  | Percentage | 78.9% | 21.1% | 100% |
|  | HC | Count | 25 | 13 | 38 |
|  |  | Percentage | 65.8% | 34.2% | 100% |
| Total |  | Count | 77 | 37 | 114 |
|  |  | Percentage | 67.5% | 32.5% | 100% |

**Note.** Counts and row percentages are shown. Percentages are calculated within each trait group (row totals = 100%). The association between trait group and adaptation class membership was evaluated using a *χ²* test in the main text. HSA = high social anhedonia group; SD-noHSA = subclinical depression without high social anhedonia group; HC = healthy control group.

**Table S7** Repeated Measures ANOVA Results for ‘Wanting’ and ‘Liking’ Across Effort-Reward Conditions

|  |  | Subclinical Depression Group  (N=30) | | Control Group (N=30) | |  |  |
| --- | --- | --- | --- | --- | --- | --- | --- |
| Condition | Dependent Variable | Mean | SD | Mean | SD | F(1,58) | *p* |
| Effort-Reward Balance (A) | ‘wanting’ | 5.01 | 0.14 | 5.88 | 0.14 | 20.146 | <.001 |
|  | ‘liking’ | 5.14 | 0.13 | 5.69 | 0.13 | 8.939 | .004 |
| Effort > Reward Imbalance (B) | ‘wanting’ | 4.21 | 0.18 | 4.95 | 0.18 | 8.522 | .005 |
|  | ‘liking’ | 2.47 | 0.19 | 2.54 | 0.19 | .071 | .790 |
| Effort < Reward Imbalance (T) | ‘wanting’ | 4.93 | 0.19 | 6.04 | 0.19 | 17.866 | <.001 |
|  | ‘liking’ | 5.74 | 0.13 | 6.57 | 0.13 | 19.860 | <.001 |

**Note.** Values are estimated marginal means (SE/SD; use the one consistent with the table). ‘Liking’ ratings are reverse-coded such that higher values indicate greater liking. A = effort–reward balance; B = effort > reward imbalance; T = effort < reward imbalance. Reported F(1,58) and p values correspond to the group effect (subclinical depression vs. control) within each condition for the specified dependent variable.

**Table S8** Comparison of Reward Motivation Adaptation Indicators (*β*_1_) for ‘Wanting’ and ‘Liking’ Between SD and HC Groups

|  | SD (N=30) | | HC (N=30) | |  |  |  |  |
| --- | --- | --- | --- | --- | --- | --- | --- | --- |
|  | Mean | SD | Mean | SD | t | df | p | Cohen’s d |
| *β*_1,_ _‘wanting’_ | 0.23 | 0.32 | 0.37 | 0.30 | -1.659 | 58 | .103 | -.428 |
| *β*_1, ‘liking’_ | 0.74 | 0.22 | 0.84 | 0.19 | -1.948 | 58 | .056 | -.503 |

**Note.** *β*_1_ represents the individual-level slope (sensitivity) of ratings (‘wanting’ or ‘liking’) as a function of incentive level across blocks/conditions; higher *β*_1_ indicates stronger scaling of subjective ratings with incentive. Independent-samples t tests compare *β*_1_ between the subclinical depression and control groups. Cohen’s d is computed as (SD − HC), such that negative values indicate lower *β*_1_ in the subclinical depression group. SD = subclinical depression group; HC = healthy control group.

**
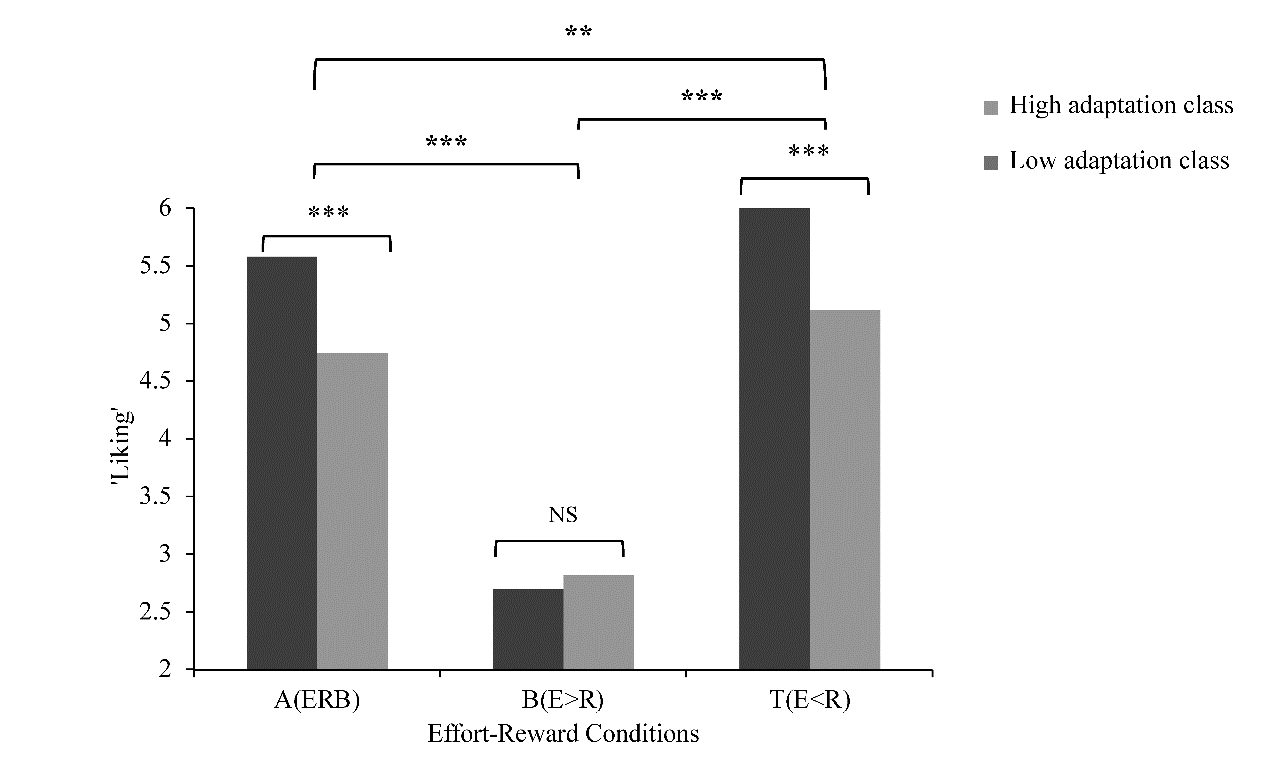
Figure S1** Behavioral Validation of High and Low Adaptation Classes Based on ‘Liking’

**Note.** Mean ‘liking’ ratings across the three effort–reward conditions are shown for the high-adaptation group (N = 77) and the low-adaptation group (N = 37). ‘Liking’ ratings are reverse-coded such that higher values indicate greater liking. Error bars represent ± SEM. A = effort–reward balance; B = effort > reward imbalance; T = effort < reward imbalance. Corresponding descriptive statistics and condition-wise group comparisons are reported in Table S5.

**References**

Franken, I. H. A., Rassin, E., & Muris, P. (2007). The assessment of anhedonia in clinical and non-clinical populations: Further validation of the Snaith–Hamilton Pleasure Scale (SHAPS). *Journal of Affective Disorders*, *99*(1–3), 83–89. https://doi.org/10.1016/j.jad.2006.08.020

Snaith, R. P., Hamilton, M., Morley, S., Humayan, A., Hargreaves, D., & Trigwell, P. (1995). A Scale for the Assessment of Hedonic Tone the Snaith–Hamilton Pleasure Scale. *British Journal of Psychiatry*, *167*(1), 99–103. https://doi.org/10.1192/bjp.167.1.99
